# The Efficacy of Thumb Spica Casting With or Without Corticosteroid Injection for De Quervain's Tenosynovitis

**DOI:** 10.7759/cureus.65408

**Published:** 2024-07-26

**Authors:** Luqman Khan, Muhammad Abdullah, Ubaid Ullah, Zeeshan Haider, Arsalan Shah Roghani, Rao E Hassan, Muhammad Waqar Khan, Adnan Ahmad, Hassamullah Khan, Waheed Ahmad

**Affiliations:** 1 Orthopaedics and Trauma, Khyber Teaching Hospital MTI, Peshawar, PAK; 2 Orthopaedics and Trauma, Khyber Teaching hospital MTI, Peshawar, PAK; 3 General Surgery, Hayatabad Medical Complex MTI, Peshawar, PAK; 4 Orthopaedics and Trauma, Hayatabad Medical Complex MTI, Peshawar, PAK

**Keywords:** “efficacy”, casting, de quervain disease, de quervain's tenosynovitis, methylprednisolone acetate injection, corticosteroids injection, thumb spica casting

## Abstract

Background and objective

De Quervain's tenosynovitis is a highly prevalent wrist pathology primarily caused by chronic thumb overuse. Its management typically begins with conservative methods, progressing to corticosteroid injections or surgery if necessary. This study compares the efficacy of thumb spica casting plus corticosteroid injection versus casting alone for treating De Quervain's tenosynovitis.

Materials and methods

This quasi-experimental study was conducted at the Department of Orthopaedics, Khyber Teaching Hospital, Peshawar, and enrolled adults aged 18-50 who presented with De Quervain's tenosynovitis. Patients were assigned to receive either corticosteroid injection plus thumb spica cast (Group A) or thumb spica cast alone (Group B). The primary outcome assessed the treatment success rate, while the secondary outcome evaluated the treatment effectiveness using visual analog scale (VAS) scores and Quick Disabilities of Arm, Shoulder, and Hand (QuickDASH).

Results

Of the initial 65 patients enrolled, 61 completed the study. Group A demonstrated a significantly higher treatment success rate (83.9%, n=26) compared to Group B (40%, n=12) (p<0.001). Pain reduction, as measured by VAS, was markedly greater in Group A (8.4 ± 1.0 to 0.4 ± 0.5) than in Group B (9.0 ± 0.8 to 5.9 ± 1.3) (p<0.001). Similarly, functional improvement assessed by QuickDASH favored Group A (89.6 ± 8.2 to 8.9 ± 6.8) over Group B (84.3 ± 10.1 to 49.1 ± 12.3) (p<0.001). No serious adverse effects related to treatments were noted in either of the groups.

Conclusions

This study supports the superiority of thumb spica casting along with local corticosteroid injection over casting alone for treating De Quervain's tenosynovitis. The combined approach led to significantly better pain relief and functional outcomes, highlighting its effectiveness as a treatment option despite the positive outcomes observed with casting alone.

## Introduction

De Quervain's tenosynovitis, also called gamers’ thumb or mother's thumb, is a common pathological condition of the wrist [1]. It is most commonly caused by chronic overuse of the thumb, attributed to myxoid degeneration with fibrous tissue deposits and increased vascularity rather than acute inflammation of the synovial lining of the extensor pollicis brevis (EPB) tendon and the abductor pollicis longus (APL) tendon [2]. The condition is more commonly found in perimenopausal and pregnant women [2]. The diagnosis is often clinical, and the signs and symptoms include pain, tenderness at the first dorsal compartment, and a positive Finkelstein test [3,4].

De Quervain's tenosynovitis is typically managed conservatively first. This might involve rest, pain relievers, and splinting or casting to immobilize the thumb [2,3]. Splinting with a thumb spica brace may offer patients temporary relief, but failure and recurrence are often high and compliance low [5]. While casting offers limited benefits on its own, research suggests that it can be combined with corticosteroid injections for better outcomes [6-8]. Corticosteroid injections are a preferred option for faster symptom relief compared to other non-surgical methods, but they do carry potential side effects [9,10]. If conservative measures fail, surgery is an option, with a high success rate but also potential risks and costs [11]. The debate on the optimal approach continues, with no clear consensus on whether conservative management, corticosteroid injections, or surgery provide the best outcomes.

Akhtar et al. have demonstrated that combining corticosteroid injection with a thumb spica cast achieved an efficacy rate of 85.1% compared to 37.4% with casting alone for De Quervain's tenosynovitis [8]. This study aimed to compare the effectiveness of these two approaches: corticosteroid injection plus thumb spica cast versus casting alone. We hypothesized that the success rate and functional outcomes would differ between these two treatment methods.

## Materials and methods

This quasi-experimental study investigated patients treated for radial side wrist pain at the Department of Orthopaedics, Khyber Teaching Hospital, Peshawar, between June 10, 2022, and December 10, 2022. Adults aged 18-50 years experiencing pain on the radial side of the wrist, with tenderness over the styloid process in the first dorsal extensor compartment and a positive Finkelstein test, were included. Patients with a history of local corticosteroid injection or oral/local NSAID treatment within the previous six months, wrist fracture, pregnancy, any diagnosed musculoskeletal disorder (e.g., rheumatoid arthritis, radiculopathy), previous surgery, severe trauma, or hypersensitivity/contraindication to lidocaine/methylprednisolone acetate were excluded.

A WHO Sample Size Calculator determined that a minimum of 19 patients per group was needed to detect an expected efficacy difference of 85.1% between corticosteroid injection with a thumb spica cast (Group A) and casting alone (Group B) for De Quervain's tenosynovitis [8]. The calculations assumed a 5% risk of type I error, a 20% risk of type II error, a bilateral test, and a 20% dropout rate. Using a non-probability consecutive sampling technique, 77 eligible patients were identified. Twelve declined participation, leaving 65 patients. These were divided into a steroid-spica group (Group A; n=33) and a casting-alone group (Group B; n=32). After obtaining approval from the Khyber Medical College Institutional Research and Ethical Review Board (IREB), patients meeting the inclusion criteria were recruited from the Department of Orthopaedics. Informed consent was obtained from all participants. Baseline demographics, including age, gender, duration of symptoms, affected and dominant hand, and occupation (unemployed, manual work, non-manual work), were recorded.

Two consultant orthopedic surgeons treated patients according to their assigned group. Group A received an injection of 40mg methylprednisolone acetate (1cc) mixed with 1-2cc of 2% lidocaine in the first dorsal compartment at the point of maximum tenderness. A 27-gauge insulin needle was used for the injection. After sterilizing the skin, the needle was inserted at a 45-degree angle along the tendons of the EPB and APL until a slight resistance was felt, then withdrawn slightly before injecting the solution. A thumb spica cast was applied afterward. Group B received only a thumb spica cast on the affected thumb. Patients in both groups were advised to minimize physical activity and prioritize rest. No pain medication was prescribed. After three weeks, the casts were removed, and patients were encouraged to resume wrist and finger movement. Clinical examinations were conducted three weeks later to assess early response, followed by three-weekly evaluations for 12 weeks.

The primary outcome measure was to achieve complete symptom resolution defined as no pain in the radial wrist, no tenderness in the thumb area at the first dorsal compartment, and a negative Finkelstein test at all follow-up appointments, until the final evaluation at 12 weeks. All three criteria needed to be negative for successful treatment; if even one remained positive, the treatment would be deemed unsuccessful. Secondary outcomes included pain intensity and functional outcome. Pain was assessed using a visual analog scale (VAS) ranging from 0 (no pain) to 10 (severe pain) before treatment and at all follow-up visits. The Quick Disabilities of the Arm, Shoulder, and Hand (QuickDASH) score was used to evaluate functional outcomes at the same time points. Data were collected on a specifically designed proforma.

Statistical analysis was performed using IBM SPSS Statistics for Windows, V. 24.0 (IBM Corp., Armonk, NY). For qualitative variables, frequencies and percentages were calculated. Quantitative variables were presented as mean ± standard deviation (SD). Data were summarized using tables and charts. Normally distributed variables were analyzed using repeated measures analysis of variance (ANOVA), while non-parametric data were analyzed using the Mann-Whitney U test. The independent samples t-test and chi-square test were used to assess the similarity of baseline characteristics between the two groups. A paired t-test was used to compare the secondary outcomes within each group. Moreover, the chi-squared test was used to assess statistical significance within each subgroup. A p-value ≤0.05 was considered statistically significant.

## Results

Sixty-five patients were enrolled in this study, with 33 assigned to Group A and 32 to Group B. Two patients from each group were lost to follow-up and hence excluded from the final analysis. Baseline characteristics, including demographics, dominant hand, affected hand, and occupation, were similar between the two groups (Table 1).

**Table 1:** Demographic Information and baseline assessment scores The independent sample t-test was used for quantitative variables with a p-value ≤0.05 considered significant. The chi-square test was used for qualitative variables with a p-value ≤0.05 deemed as significant QuickDASH: Quick Disabilities of the Arm, Shoulder, and Hand; SD: standard deviation; VAS: visual analog scale

Characteristics		Steroid + spica cast	Casting alone	Total	P-value
Number of patients		31	30	61	
Age, years, mean ± SD		32.2 ± 5.5	30.6 ± 4.9	32.1 ± 6.3	>0.05
Gender, male/female		6/25	7/23	13/48	>0.05
Duration of symptoms, months, mean ± SD		2.4 ± 0.6	2.7 ± 0.8	2.5 ± 0.7	>0.05
Dominance, right/left		24/7	23/7	47/14	>0.05
Affected hand, right/left		23/8	25/5	48/13	>0.05
Occupation	Unemployed	3	5	8	>0.05
	Manual work	19	17	36	
	Non-manual work	9	8	17	
VAS score before treatment, mean ± SD		8.4 ± 1.0	9.0 ± 0.8	8.7 ± 0.9	>0.05
QuickDASH score before treatment, mean ± SD		89.6 ± 8.2	84.3 ± 10.1	84.05 ± 9.2	>0.05

The treatment was significantly more effective in Group A (corticosteroid injection with thumb spica cast) compared to Group B (thumb spica cast alone). In Group A, 83.9% (26 patients) achieved successful outcomes, compared to only 40% (12 patients) in Group B (p<0.001). No injection or spica cast-related complication was observed during the study period in any of the two groups. Detailed results for pain and function are presented in Table 2. VAS scores significantly decreased from pre-treatment to the three-month follow-up visit in both groups. However, the reduction was much greater in Group A (8.4 ± 1.0 to 0.4 ± 0.5) compared to Group B (9.0 ± 0.8 to 5.9 ± 1.3) (p<0.001). This translates to a 95% and 34% reduction in pain scores, respectively. Similarly, the QuickDASH score, which assesses functional limitations, showed a significantly greater improvement in Group A (89.6 ± 8.2 to 8.9 ± 6.8) compared to Group B (84.3 ± 10.1 to 49.1 ± 12.3) (p<0.001). This represents an 82.5% and 42% reduction in functional limitations, respectively.

**Table 2:** Change in VAS and QuickDASH scores by treatment group The paired t-test was used with a p-value ≤0.05 deemed significant CS + cast: corticosteroid injection plus thumb spica casting; QuickDASH: Quick Disabilities of the Arm, Shoulder, and Hand; SD: standard deviation; VAS: visual analog scale

Variables		N	Mean ± SD	t-test	Effect size	P-value
VAS score in Group A (CS + cast)	Before treatment	31	8.4 ± 1.0	38.3	6.99	<0.001
	After treatment	31	0.4 ± 0.5			
VAS score in Group B (cast alone)	Before treatment	30	9.0 ± 0.8	10.6	1.94	<0.001
	After treatment	30	5.9 ± 1.3			
QuickDASH score in Group A (CS + cast)	Before treatment	31	89.6 ± 8.2	49.9	8.97	<0.001
	After treatment	31	8.9 ± 6.8			
QuickDASH score in Group B (cast alone)	Before treatment	30	84.3 ± 10.1	14.6	2.67	<0.001
	After treatment	30	49.1 ± 12.3			

Stratification of efficacy in both groups concerning age, gender, and duration of complaints is presented in Table 3.

**Table 3:** Treatment effect by age, gender, and disease duration The chi-square test and Fisher's exact test were used with a p-value ≤0.05 deemed significant CS + cast: corticosteroid injection plus thumb spica casting

Variables			Efficacy		P-value
			Yes	No	
Age groups, years	18-30	CS + cast	16	3	0.001
		Cast clone	5	11	
	31-50	CS + cast	10	2	0.001
		Cast alone	7	19	
Gender	Male	CS + cast	6	1	0.55
		Cast alone	4	2	
	Female	CS + cast	20	4	0.000
		Cast alone	8	16	
Duration of the disease, months	1-3	CS + cast	18	3	0.003
		Cast alone	8	11	
	>3	CS + cast	8	2	0.043
		Cast alone	4	7	

## Discussion

De Quervain's tenosynovitis often becomes chronic due to repetitive hand use, thereby hindering the healing process. The unclear pain mechanism can also complicate the treatment selection [12]. While no single treatment has been definitively proven to be optimal, research suggests that corticosteroid injections, with an 83% success rate, are the most effective option [13]. Our study findings align with those of prior trials, supporting the superiority of local corticosteroid injections plus thumb spica casting for De Quervain's tenosynovitis [6-8,12-14].

The average age of our participants was 32.1 ± 6.3 years, with a mean pre-treatment VAS score of 8.7 ± 0.9 and QuickDASH score of 84.05 ± 9.2. Mehdinasab and Alemohammad have reported similar participant ages (32.83 ± 8.9 years for combined treatment, 29.61 ± 7.7 years for cast alone). Their study used a 0-100 VAS scale (0=no pain, 100=worst pain), finding pre-treatment scores of 97.16 ± 2.31 (combined) and 95.89 ± 3.31 (cast alone) [7]. Mardani-Kivi et al. reported pre-treatment scores of 8.7 ± 1.0 (VAS) and 84 ± 10 (QuickDASH) for De Quervain's tenosynovitis treated with corticosteroid injection with or without a cast [6]. Akhtar et al. also investigated this, finding pre-treatment VAS scores of 4.55 ± 1.71 (combined) and 4.45 ± 1.01 (cast alone), with QuickDASH scores of 46.75 ± 14.84 (combined) and 42.01 ± 13.8 (cast alone) [8].

Our results support the findings of other studies. Mardani-Kivi et al. reported a success rate of 93% with combined steroid injection and spica cast, compared to only 69% success with steroid injection alone [6]. Two recent studies on De Quervain's disease treatment with contrasting results were analyzed. A Nepalese study found an 89.7% success rate with corticosteroid injection and spica cast, compared to 50% success with injection alone [12]. Conversely, a Pakistani study reported lower efficacy with combined treatment (70.7%) than with injection alone (82.9%) [13]. Studies similar to our design from India and Pakistan also reported higher success rates with combined treatment (100% and 85.1%, respectively) compared to cast alone (35% and 37.4%, respectively) [8,14]. Our study showed an 83.9% success rate with combined treatment and 40% with cast immobilization alone. A recent systematic review of 30 De Quervain's tenosynovitis studies concluded that corticosteroid injection with thumb spica immobilization is likely the most effective short- and mid-term treatment for function [15]. This supports our findings, as the group receiving this combined therapy showed significantly improved primary outcomes as well as better pain and functional outcomes profiles.

Our study found significant improvements in pain and function in the steroid plus spica group compared to casting alone. VAS scores improved to 0.4 ± 0.5 in the steroid plus spica group versus 5.9 ± 1.3 in the casting group. Similarly, QuickDASH scores improved to 8.9 ± 6.8 in the steroid plus spica group compared to 49.1 ± 12.3 in the casting group. Akhtar et al. found similar results: the VAS score significantly improved in the steroid plus spica group (0.79 ± 1.17) compared to the casting alone group (2.53 ± 1.67). The QuickDASH score improved to 11.7 ± 9.5 in the steroid plus spica group and 28.25 ± 18.71 in the casting alone group [8]. Likewise, Mehdinasab and Alemohammad reported a greater improvement in the VAS score with the combined treatment (6.70 ± 6.82) compared to casting alone (17.3 ± 11.34) [7].

Our study has a few limitations, primarily its quasi-experimental nature and single-center design, which limit the generalizability of its findings. Studies employing a multicenter design with a larger sample size could assess long-term outcomes more effectively. Additionally, the lack of a validated functional outcome measure restricted our evaluation. Future trials should address these limitations, exploring various dosages and combinations of steroid injections with spica casts and casting alone.

To sum up, our study suggests that corticosteroid injections combined with thumb spica casting offer the most effective treatment for De Quervain's tenosynovitis, in line with previous research. This approach significantly improved pain and function scores compared to casting alone.

## Conclusions

Our findings reveal the superiority of thumb spica casting along with corticosteroid injection inside the combined synovial lining of EBL and ABL over casting alone for treating De Quervain's tenosynovitis. This combined treatment approach demonstrated significantly improved pain and function compared to casting alone, with good patient compliance and minimal complications, although casting alone also yielded positive results, albeit with a lower success rate.
